# Pulmonary Embolism in Transit Across a Patent Foramen Ovale

**DOI:** 10.7759/cureus.23026

**Published:** 2022-03-10

**Authors:** Taylor J Manes, Zain Mohiuddin, Michael Bage

**Affiliations:** 1 Cardiology, A.T. Still University, Kirksville College of Osteopathic Medicine, Kirksville, USA; 2 Internal Medicine, Western Reserve Hospital, Cuyahoga Falls, USA; 3 Cardiology, Western Reserve Hospital, Cuyahoga Falls, USA

**Keywords:** thrombolysis, mcconnell’s sign, patent foramen ovale, thrombus, extracorporeal membrane oxygenation, pulmonary embolus, heart failure, syncope, dyspnea, saddle pulmonary embolus

## Abstract

A pulmonary embolism (PE) is an obstruction in a pulmonary artery, and a saddle PE occurs when the obstruction is lodged in the main pulmonary trunk and spans the left and right pulmonary arteries. The current case study describes complications of a thrombus in transit across a patent foramen ovale (PFO). A 35-year-old female presented to the emergency department after a nontraumatic syncopal fall. She had recently returned from a cross-country flight 10 days before and had noticed left calf tenderness when exiting the plane. Vitals were notable for sinus tachycardia at 120 bpm. An electrocardiogram indicated an S1Q3T3 pattern, and chest computed tomographic angiography was positive for a saddle PE. A 2D (two-dimensional) transthoracic echocardiogram showed right ventricular free wall hypokinesis and McConnell’s sign. Echocardiogram findings were concomitant with a thrombus in transit across the interatrial septum, indicating a possible PFO. An emergency pulmonary embolectomy with cardiopulmonary bypass and closure of her PFO was performed the following morning and complicated by cardiogenic shock and subsequent cardiac arrest. The patient was resuscitated in the operating room but failed to be removed from cardiopulmonary bypass, requiring low-dose inotropic support and venoarterial extracorporeal membrane oxygenation flow at 4 L/min. After a repeat right pulmonary artery thrombectomy and two subsequent transesophageal echocardiograms indicated stable right ventricular systolic function, decannulation was performed. The patient was discharged on day 17 with long-term anticoagulation and home healthcare. In the current case report, the patient’s unstable and deteriorating condition was complicated by unusual findings of a thrombus in transit across a PFO. These additional echocardiogram findings represented an unusual case that warranted surgical treatment instead of systemic thrombolysis therapy because of the increased risk of systemic clot embolization.

## Introduction

A pulmonary embolism (PE) is an obstruction in the pulmonary artery caused by tissue or air. These obstructions are the third most common cause of mortality in hospitalized patients, and a saddle PE is associated with an increased clot burden that may lead to right ventricular (RV) dysfunction [1]. Studies have shown that saddle PEs are a significant risk factor for in-hospital mortality and are associated with early death [2,3]. In general, massive and submassive PEs cause approximately 100,000 to 180,000 deaths each year in the United States [4]. Although about 50% of patients with a PE are asymptomatic, symptomatic patients may present with a variety of symptoms, such as dyspnea, pleuritic chest pain, cough, syncope, tachypnea, and tachycardia [5]. Because most of these findings are nonspecific, additional clinical tests are necessary for proper diagnosis. Established methods to evaluate the likelihood of a PE include both the Wells and Geneva scoring systems, venous duplex ultrasound, and D-dimer laboratory tests [4]. Computed tomographic (CT) pulmonary angiography is the gold standard for imaging, but ventilation-perfusion scans can also be used when CT scans are contraindicated [6].

Treatment of massive and submassive PEs depends on the patient’s clinical state. Research suggests catheter-directed thrombectomy (CDT) has better outcomes than systemic thrombolysis [7,8]. Further, systemic thrombolysis has been associated with bleeding and hemorrhagic stroke [8,9], whereas CDT has been shown to improve RV function [8]. Therefore, CDT should be considered for patients with a massive PE when thrombolysis therapy has failed or is contraindicated and for patients in shock who may die before systemic thrombolysis takes effect [9]. Because PEs are associated with serious long-term consequences when not immediately treated, surgical embolectomy, which has the lowest rate of stroke, is preferred for an intracardiac thrombus, especially since thrombolysis is associated with a higher mortality rate [10]. The current case study describes complications of a thrombus in transit across a patent foramen ovale (PFO). Because of a risk of systemic clot embolization for this patient, the standard use of CDT or pharmacologic thrombolysis was limited.

## Case presentation

**Figure 1:**
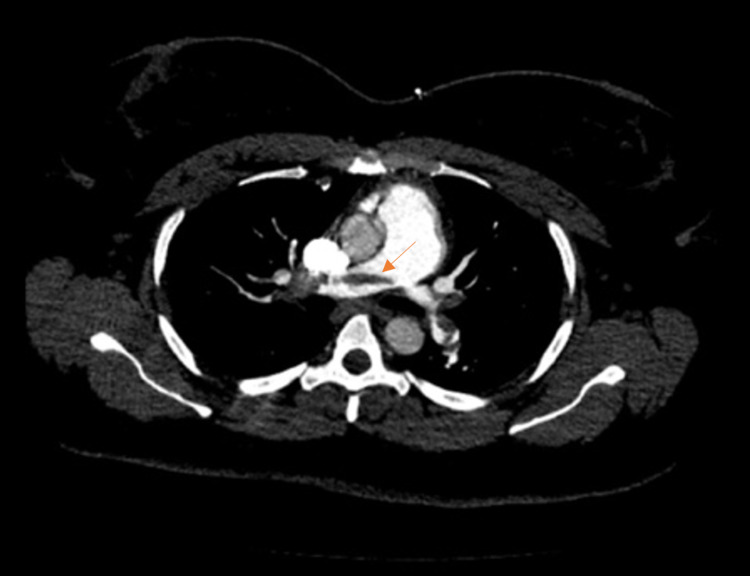
Computed tomographic angiography of the chest (axial view) Computed tomographic angiography of the chest demonstrating a large saddle embolus (orange arrow) traversing both right and left main pulmonary arteries with additional emboli distally.

**Figure 2:**
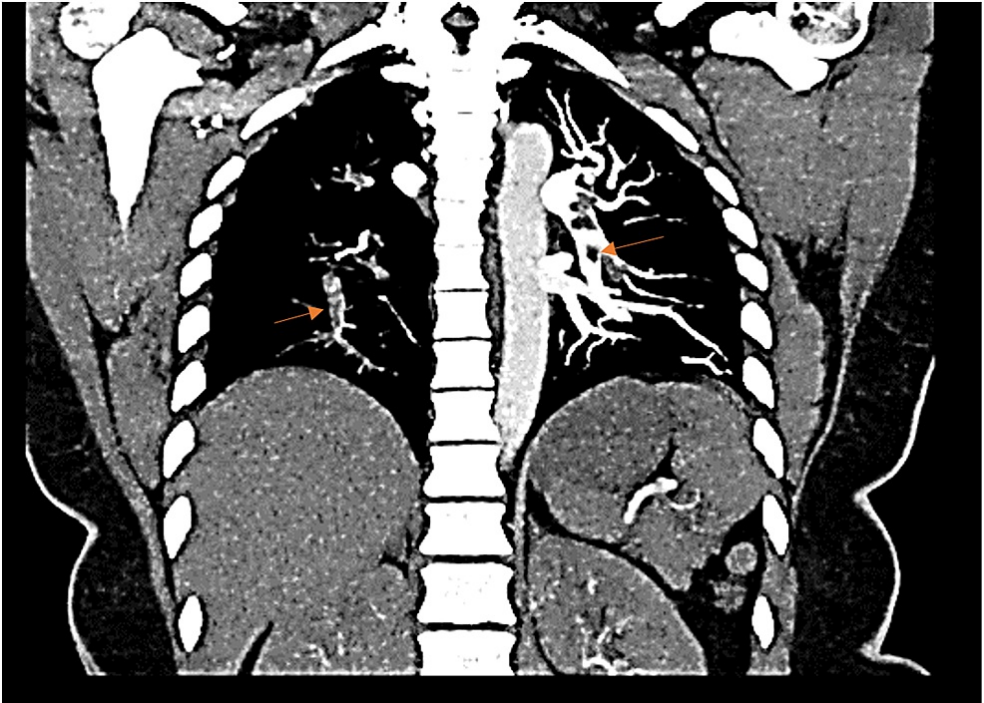
Computed tomographic angiography of the chest (coronal view). Computed tomographic angiography of the chest showing multiple emboli (orange arrows) in the upper and lower lobes bilaterally.

A 35-year-old obese African American, a non-smoking female, presented to the emergency department after a nontraumatic syncopal fall. She appeared pale with signs of diaphoresis and dyspnea. The patient reported that she had recently returned from a cross-country flight 10 days before and had noticed left calf tenderness when exiting the plane. She was currently on oral contraceptive therapy and denied a previous history of deep vein thrombosis or PE. Her vitals were notable for sinus tachycardia at 120 bpm, and her blood pressure was 141/81 mmHg. Oxygen saturation was 97% on room air, and her respiratory rate was normal at 14 breathes per minute. High-sensitivity troponin T was elevated at 59 ng/mL. An initial electrocardiogram showed sinus tachycardia with an S1Q3T3 pattern. Based on the presentation, a PE was suspected. A CT angiography indicated a large saddle embolus traversing both rights and left main pulmonary arteries with multiple emboli in the upper and lower lobes bilaterally (Figures 1, 2). 

**Figure 3:**
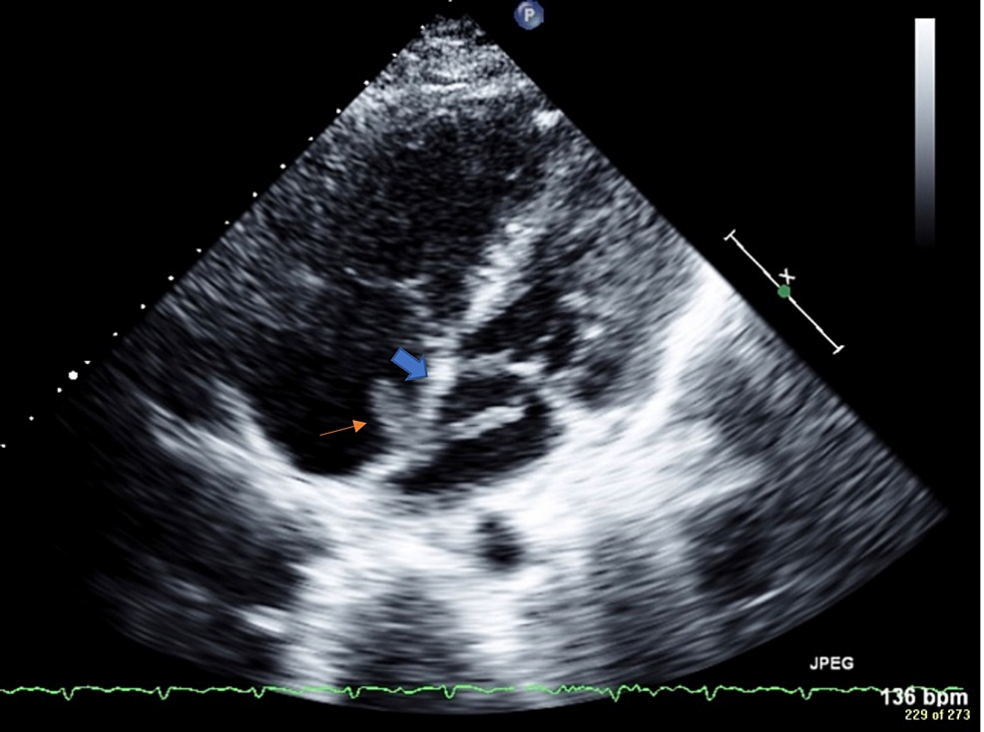
Two-dimensional transthoracic echocardiogram with color flow and Doppler ultrasonography. Two-dimensional transthoracic echocardiogram showing a thrombus (orange arrow) in transit lodged across the interatrial septum (thick blue arrow). One lobe is present in the right atrium, measuring 1.4 cm × 0.6 cm, and one lobe is in the left atrium, measuring more than 1 cm in length.

The patient was immediately started on a heparin drip. A 2D (two-dimensional) transthoracic echocardiogram was performed and showed an ejection fraction of 56%. Consistent with McConnell’s sign, free wall hypokinesis of the RV was noted in the presence of normal apical contractility. No clear PFO was noted by color Doppler ultrasonography, but there was a mobile mass on the right and left sides of the atrial septum (Figure 3), which suggested a thrombus in transit lodged across the interatrial septum. The mass in the right atrium measured 1.4 cm × 0.6 cm. There were two lobes in the left atrium that each measured more than 1 cm in length. The origin of the thrombus was uncertain but likely migrated from the peripheral venous system within the lower extremities.

The patient was transferred to a tertiary care center, and a cardiothoracic surgeon performed an emergent pulmonary embolectomy with cardiopulmonary bypass and closure of the PFO. Intraoperatively, the surgeon confirmed a PFO and created a large atrial septal defect within the fossa ovalis to explore the left atrium. At this time, a transesophageal echocardiogram (TEE) was performed and verified a PFO with left-to-right shunting of blood as identified by color Doppler ultrasonography. This mechanism of shunting was likely secondary to the creation of an atrial septal defect within the fossa ovalis. Findings were consistent with acute right heart failure, including moderate tricuspid regurgitation, severely dilated right atrium, significant pulmonary hypertension, and a small pericardial effusion. Because of her worsening condition, a transverse fashion pulmonary arteriotomy was performed, removing a 0.5-cm wide by a 20-cm long piece of clot residing within the right and left pulmonary arteries. Both pulmonary arteries were suctioned to aid the removal of the emboli. The patient then went into cardiogenic shock and later cardiopulmonary arrest. She was successfully resuscitated in the operating room but failed to be removed from cardiopulmonary bypass after three attempts, resulting in right heart dilation and acute right heart failure. Postoperatively, the patient required low-dose inotropic support and venoarterial extracorporeal membrane oxygenation at 4 L/min. On day five, there was evidence of persistent right heart failure despite the previous pulmonary embolectomy. Therefore, a repeat right pulmonary artery thrombectomy was performed, again retrieving a large amount of clot. After this procedure, the patient’s left and right systolic function continuously improved on subsequent echocardiogram studies. Another TEE was performed on day 10 and indicated hyperdynamic left ventricular systolic function with an ejection fraction of 70%-75% and improved RV function. Decannulation of the extracorporeal membrane oxygenation was performed the next day, and her condition continued to improve. She was discharged on day 17 with apixaban as a chronic anticoagulation therapy.

## Discussion

Because PEs are a common cause of death in hospitalized patients [1], recognition and management of this condition need to occur rapidly. Further, several comorbidities may contribute to associated risks of developing a PE. According to American Heart Association guidelines, PEs are classified as massive, submassive, or low-risk [10]. More specifically, a massive PE is defined as sustained hypotension requiring inotropic support or persistent bradycardia with signs of shock [10]. A submassive PE is defined as unremarkable blood pressure with either RV dysfunction or myocardial necrosis, and a low-risk PE is an acute PE that does not fit the criteria for a massive or submassive PE [10]. As expected, massive PEs are associated with the highest risk of mortality at 25%-65% [10]. Therefore, rapid triage and classification of patients with PE are necessary to identify those most likely to deteriorate, allowing for more rapid treatment. Pulmonary embolism response teams (PERT) have been implemented for this reason and include multiple specialists in cardiology, emergency medicine, pulmonary medicine, and critical care [11]. Reducing the time to stratify and treat hospitalized patients with a PE is crucial. For example, studies have found that reducing the onset of anticoagulation directly impacts mortality for patients with an acute PE [12,13]. Studies have also found increased time before anticoagulation is a risk factor for increased mortality [4]. However, in one of those studies [12], PERT was associated with a reduction in 30-day inpatient mortality and major bleeding.

Typically, in stable patients, anticoagulation therapy is initiated immediately. The use of direct oral anticoagulants (DOACs) and vitamin K antagonists are common in PE management [10]. Examples of DOAC medications include rivaroxaban, with a prescribed oral dosage of 15 mg twice daily, or apixaban, with a prescribed oral dosage of 10 mg twice daily [10]. When DOACs are inappropriate for the patient, low-molecular-weight heparin, intravenous unfractionated heparin, or fondaparinux are commonly used [10].

In unstable patients, the initial treatment includes stabilization of the patient’s vitals by treating shock or persistent hypotension [10]. Thrombolytic therapy, catheter-directed lysis, and surgical pulmonary embolectomy are treatments that have been used for high-risk patients. For example, in patients without bleeding risk, thrombolytic therapy, such as tissue plasminogen activator, is recommended [10]. In patients with high bleeding risk, systemic thrombolysis should be avoided because of potential hemorrhage [14]. For patients with an increased risk of bleeding or who have failed systemic thrombolysis, catheter-directed lysis therapy is recommended [10]. Surgical pulmonary embolectomy is another treatment option that can be used when systemic thrombolysis fails and the patient’s clinical condition continues to deteriorate [10]. 

The patient in the current case study had a submassive PE with RV dysfunction and electrocardiogram abnormalities. She showed clinical signs of deterioration despite treatment with intravenous unfractionated heparin, and there were signs of a clot in transit within the PFO. A surgical pulmonary artery embolectomy was performed based on the patient’s rapid clinical deterioration and the unusual intracardiac thrombus. After the procedure, the patient continued to demonstrate signs of a high-risk PE, requiring the use of extracorporeal membrane oxygenation and low-dose inotropic support. A second surgical pulmonary artery embolectomy was then performed, after which the patient’s condition improved.

## Conclusions

Pulmonary embolisms increase mortality in patients and can cause long-term complications. When evaluating a patient with symptoms of dyspnea that progressively worsen, medical providers should consider a PE. In conjunction with PERT, better education and recognition of acute PE will result in rapid care and better clinical outcomes for these patients. The current report described a rare case of a 35-year-old female with a submassive PE complicated by a thrombus in transit across a PFO that warranted a surgical pulmonary embolectomy instead of systemic thrombolysis therapy. Increased awareness of this type of clinical presentation and the common strategies used for risk stratification and management of PEs may improve outcomes and reduce mortality.
